# Access to Healthcare in Brazilian Prisons: Why is it Important to Look at the Bureaucracy and Policy Implementation?

**DOI:** 10.3389/ijph.2023.1605266

**Published:** 2023-01-12

**Authors:** Mariana Scaff Haddad Bartos

**Affiliations:** Department of Politics, Management and Health, School of Public Health, University of São Paulo, São Paulo, Brazil

**Keywords:** public health policies, implementation, persons deprived of liberty, street-level bureaucracy, access to healthcare in prison, PNAISP

**The IJPH series “Young Researcher Editorial” is a training project of the Swiss School of Public Health.**

With 1,4 million people incarcerated, Latin America´s prison population has grown exponentially in recent decades, doubling its numbers since 2000 [1]. Brazil is the leading country in the region, with 835,000 inmates, holding the third-largest prison population in the world [2]. This expansion has worsened the conditions in prisons, straining their already precarious infrastructure. Overcrowding, unsanitary environments, and a lack of adequate ventilation and lighting make prisons suitable spaces for the spread of diseases. In this context, it is important to discuss the relevance and implementation of public healthcare access policies that take place within the walls.

In Brazil, public health policies for the prison system are covered by the 2014 National Policy for Comprehensive Healthcare for Persons Deprived of Liberty (PNAISP). It aims to guarantee that those deprived of liberty can still access comprehensive care within the Unified Health System (SUS). Though this policy exists, it is important to reflect on how its implementation is strongly influenced by the beliefs, ideas, and value-judgements of those responsible for enacting it in prisons.

The individuals responsible for implementing the PNAISP are multidisciplinary professionals in prison primary care teams. The organization of these teams depends on factors such as the number of people incarcerated in the prison unit and their epidemiological profiles. At a minimum, a team must include a doctor, a nurse, a nursing technician or nursing assistant, a dental surgeon, and an oral health technician or assistant. Larger teams may also add psychologists, social workers, nutritionists, and physical therapists.

Team professionals are street-level bureaucrats. They are mediating agents between the state and its citizens [3] with direct interaction with those affected by the prison healthcare policies. Their performance is influenced by political, economic, and institutional conditions, along with their own biases, interests, and ideologies [4]. When they implement public policies following their values, beliefs, and ideals, street-level bureaucrats may deviate from the intent of the policymakers [5].

Several studies on bureaucracy support this phenomenon. For example, a study of community health agents in Brazil [5] pointed out that their various trajectories and personal issues could change the dynamics underlying the processes of health policy implementation. The contextual conditions were highly determinant, and people, values, and references also influenced implementation. Similarly, a study of the Family Allowance Fund in France [6] found that daily interactions between the fund agents and the families that received the benefits were influenced by the individual social agency of bureaucrats who decided how to implement public policy.

Because bureaucrats’ personal references shape the implementation of PNAISP and similar policies, we must look beyond formal policy specifications to improve prison healthcare. Institutional rules and directives are often abstract and can be broadly interpreted, which gives the implementing bureaucrat much freedom of action. When bureaucrats base their actions on ethical judgments influenced by their own agendas, vision, and world values, they act under value-oriented discretion, as Taylor and Kelly have coined [7]. Looking at the implementation of health policies in complex environments and contexts, such as prisons, requires recognizing the performance of street-level bureaucracy beyond the idea of neutrality, command, and obedience of these bureaucrats.

In this way, if it is expected to ensure that access to health is equitable for people in vulnerable situations, including those who are deprived of their liberty, it is imperative to understand the impact that the agency of policy-implementing bureaucrats has in such contexts. Only by learning about the circumstances and mechanisms through which implementation is affected will we be able to develop successful policies and solutions that address such issues.
